# Physiotherapist-led rehabilitation for patients with chronic musculoskeletal pain: interventions and promising long-term outcomes

**DOI:** 10.1186/s12891-021-04780-x

**Published:** 2021-10-28

**Authors:** Anna Trulsson Schouenborg, Marcelo Rivano Fischer, Elisabeth Bondesson, Anna Jöud

**Affiliations:** 1 Department of Health Sciences, Division of Physiotherapy, Lund University, Lund, Sweden; 2 Department of Neurosurgery and Pain Rehabilitation, Skane University Hospital, Lund, Sweden; 3 Department of Health Sciences, Research Group Rehabilitation Medicine, Lund University, Lund, Sweden; 4 Department of Clinical Sciences Lund, Division of Orthopaedics, Lund University, Lund, Sweden; 5 Department of Laboratory Medicine, Division of Occupational and Environmental Medicine, Lund University, Lund, Sweden; 6 Department of Clinical Sciences Lund, Lund University, Lund, Sweden; 7 Department of Research and Education, Skane University Hospital, Lund, Sweden

**Keywords:** Musculoskeletal pain, Chronic pain, Physical therapy, Rehabilitation, Interventions

## Abstract

**Background:**

There is no consensus on best content, set-up, category of involved healthcare professionals or duration of rehabilitation-programs for patients with chronic musculoskeletal pain, and outcomes show varying results. Individual care regimes for sub-groups of patients have been proposed.

**Aim:**

To describe the type of interventions used in a physiotherapist-led, rehabilitation-program for patients with chronic musculoskeletal pain, refractory to preceding treatments. A second aim was to report clinical outcomes at 1-year follow-up after the intervention period.

**Methods:**

All patients referred to physiotherapist within a specialist pain-unit due to being refractory to preceding treatments, and deemed fit to undergo physiotherapy-based, individualized rehabilitation during 2014–2018 were consecutively included and followed-up 1 year after ending the program. The inclusion was based on structured ‘clinical reasoning’ using the referral, examination and on patient-relevant outcome measures. The individual interventions, recorded according to a manual used when reading the patients’ medical records, were described. Primary outcomes were clinical results of perceived pain, disability and overall health at start, discharge and 1 year after discharge.

**Results:**

In total, 274 patients (mean age 42 years, 71% women) were included, suffering from chronic, severe, musculoskeletal pain (VAS median 7/10, duration median 2.8 years) and moderate disability. The most frequent interventions were education, sensorimotor training, physical activity-advice and interventions for structures/functions (for example manual techniques, stretching) for a median of nine sessions during five months. Despite refractory to preceding treatments, 45% of the patients rated clinically important improvements on pain, 61% on disability and 50% on overall health at discharge and the figures were similar at 1-year follow-up.

**Conclusions:**

A physiotherapist-led, one-to-one, rehabilitation-program of median nine sessions during five months, combining individualized education, sensorimotor training, physical activity-advice and interventions for structures/functions rendered clinically relevant improvements on pain, disability and overall health in half of the patients at 1-year follow-up. Since the cohort consisted of patients refractory to preceding treatments, we believe that these results warrant further studies to identify the subgroups of patients with chronic musculoskeletal pain that will improve from new, distinctive, resource-effective rehabilitation-programs involving individualized rehabilitation.

## Background

Chronic musculoskeletal pain, CMP, concerns pain lasting more than 3 months [1], is common in men and women and has a weighted mean prevalence in adults of 20% [2–4]. CMP includes conditions that affects joints, bones, muscles, tendons or multiple body areas and/or components (such as regional or widespread pain) and limits mobility, function and participation [5]. Patients with CMP often report fatigue, depression and anxiety, as well as socio-economic consequences of their pain-condition [6, 7]. Moreover, CMP is known to limit the individual’s engagement in activities and societal participation, leading to a complex health situation both for the patient and the society, and a small number of these patients make repetitive visits to health care providers [8].

Treatment for early (acute/subacute) musculoskeletal pain is often delivered in primary care [9] according to clinical guidelines – as for example in the treatment of low back- or neck pain – with the aim to remove or decrease the pain [10, 11], although with varying treatment-outcomes [12, 13]. For CMP, physical and psychological combined interventions are recommended to be performed within a cognitive behavioral framework [6, 7, 14, 15]. Such combined interventions are often delivered to groups of patients by teams of health-care professionals making complementary contributions to improve the outcome, are delivered in specialized hospital units over a lengthy period of time, with the aim to increase the patients’ quality of life and ability to conduct a normal life through adequate pain management [6, 7, 14]. Such interdisciplinary pain rehabilitation has been described as time- and resource demanding both for the individual patient as well as for the healthcare system [16]. Studies on team pain rehabilitation point out that the type of interventions used vary, and are seldom described in detail [17–19]*.* Additionally, and in line with studies for acute/subacute musculoskeletal pain, outcomes vary [15, 16, 20].

In recent years, individual care regimes for sub-groups of patients with chronic pain have been proposed [21], stressing that patients’ specific needs, preferences and abilities should be considered [14], and patient-centred care are proposed to be a cornerstone for best care for patients with musculoskeletal pain [22–24]. Also, evolving research data suggests that to optimize rehabilitation in patients with CMP, treatments and exercises should also be tailored to the pain mechanisms in combination with somatic, psychosocial, cognitive, motivational and behavioral factors [22, 25]. Consequently, there is a necessity to explore alternative rehabilitation options for sub-groups of patients with CMP, preferably in a treatment-window before interdisciplinary pain rehabilitation thus demanding less resources.

To explore such an alternative rehabilitation option, a Physiotherapy Pain Rehabilitation Program, PT-PRP, was launched in 2011 at a specialized pain rehabilitation unit in southern Sweden. At that time, a number of patients were referred to the unit due to being refractory to preceding treatments (usually physiotherapeutic- and pharmacological interventions) reporting a level of complexity in their unresolved pain problems that neither matched the requirements of primary care, nor interdisciplinary pain rehabilitation. The PT-PRP was conceived as a patient-centered option for patients with CMP, requiring one-to-one physiotherapy. The underlying treatment philosophy to the PT-PRP focused on the optimization of patients’ value-based activities and participation, using physiotherapist-led-interventions (formulated in a policy statement to meet the standards of a consultative accreditation process - Commission on Accreditation of Rehabilitation Facilities, CARF [26]). The treatment-philosophy also covered supporting the patient in the process of developing flexible physical, and to some extent also mental, strategies for living an active life with CMP including how to lessen symptoms and consequences of pain, but also of for example, decreased: strength, fitness, motor control, posture or of kinesiophobia. This means that, at baseline, the rehabilitation could start with either a ‘time-contingent’ treatment approach or a ‘pain-contingent’ treatment approach (for description see [27, 28]), the latter not ruling out pain relief (completely or partly) - often practiced at the referring caring facility in for example primary care. In patients where a ‘pain-contingent’ approach was deemed appropriate at base-line, this could be continued, and if found inappropriate at a later time point - the patients were invited to explore a ‘time-contingent’ approach [27, 28], meaning that the physiotherapist-led education, exercises and the practice of pain management strategies were no longer governed only by pain, but by the main goal, namely that of increasing valued activities. Therefore, depending on patient status, interventions aiming at pain moderation and strategies for increased activities could be implemented simultaneously, or patients could switch strategies during the rehabilitation, based on their experiences. Irrespective of treatment approach, all patients’ written, value-based treatment-goals were aiming at increasing activities. Thus, the PT-PRP was intended for patients willing to explore ‘pain- and/or time-contingent’ treatment approaches with focus on value-based goals - a distinctive treatment philosophy not often performed at the same caring facility. The patients therefore could be expected to reach their activity-based goals with increased, unchanged or decreased pain. The choice of treatment approach and the interventions used in the PT-PRP were not specified in advance and were selected based on the reasoning and decision-making process used in clinical practice named ‘clinical reasoning’ as defined by [29, 30].

For management and evaluation purposes of the PT-PRP, also program-goals were expressed for the outcome of the program, describing the patients´ experiences of pain, activity and common health, as evaluated by patient-relevant outcome measures, PROMs, collected at start, discharge and 1 year after discharge, showing yearly promising clinical results. To investigate the PT-PRP content and outcomes for a larger population, all data from patients enlisted during 2014–2018 are evaluated in the present study, forming a basis for a future RCT (randomized controlled trial).

The main aim of this study was to describe the type of interventions used in a physiotherapist-led rehabilitation-program for patients with CMP refractory to preceding treatments and therefore referred to specialized care. A further aim was to report clinical outcomes at 1-year after end of the intervention period.

## Methods

### Context of the rehabilitation program

The PT-PRP was developed to meet the clinical demands of individualization but also the demands of resource efficiency and patient relevant outcomes and had recurrently been accredited according to international standards of quality, value, and outcomes through a consultative accreditation process (CARF [26]). Since the Swedish health care system ensures equal access to health care to all residing citizens, all residents in the southern part of Sweden had cost-free access to the PT-PRP.

### Inclusion and exclusion in the PT-PRP

Inclusion and exclusion of the program followed a structured, written routine based on: 1) the written referral, 2) questionnaires with PROMs, and on 3) clinical examination according to ‘clinical reasoning’ [22, 29, 30].

All referrals were screened by a senior physiotherapist who verified that the patient suffered from chronic (> 3 months) musculoskeletal pain, and that the patient was 18 years or older. Additionally, a primary screening for exclusion was performed from the referral, and then followed up during the later examination, concerning: acute psychiatric illness or acute crisis, if the patient had urgent social- or economic difficulties or was in a present alcohol- or drug abuse and if the patient had social or psychological consequences hindering the improvement of physiotherapeutic interventions. The referrals were sent mostly from primary care (either directly or via the Unit’s own interdisciplinary assessment teams where the physiotherapist was one of the team-members) or from orthopedic-, rheumatology-, oto-rhino laryngology clinics due to persisting pain-related complaints, refractory to preceding treatments at the referring caring facility.

After the referral screening, questionnaires with PROMs were sent to the patient with queries about pain, activity-limitations, pain management, perceived health and consequences in life. The PROMs were sent at start, at discharge and 1 year after discharge. For specific content; see “Assessment and questionnaires…” and “Global assessments…” below.

When the patient was examined in the PT-PRP, pain modalities (nociceptive/neuropathic/nociplastic pain), joints, muscles, nerves, functions and activities were analyzed also including the perspective of sensorimotor control in functional movements and joint stabilization [31]. Moreover, an orientation on cognitive- and emotional factors, social situation, motivation and other possible consequences of movement related pain, behavior and life was assessed, to enable tailored patient-centered interventions and education [22]. The patient informed the physiotherapist on current pharmacological treatment, and if the patient had any medication, this was managed by the physician at the referring facility during the whole PT-PRP. Previous medical records were used (on patient’s consent) for information on previous health conditions and physiotherapy interventions. Inclusion in the PT-PRP was decided in collaboration between the patient and the physiotherapist.

Thus, the physiotherapist used the combined information of the referral, the examination, the PROMs and the medical records data to decide on whether or not the patient had a complexity of pain problems that could benefit from further rehabilitation led by a physiotherapist in specialized care in a one-to-one relation (not group-rehabilitation) or if the patient required interdisciplinary pain rehabilitation instead. This decision was made according to ‘clinical reasoning’, defined as a “process in which the therapist, interacting with the patient and others (such as family members or others providing care), helps patients structure meaning, goals, and health management strategies based on clinical data, patient choices, and professional judgment and knowledge” [29], and comprises a range of ‘clinical reasoning’ skills or strategies representing a diversity of thinking and actions in a variety of tasks ranging from how to diagnose (for example pain modality and somatic/medical factors but also to consider cognitive/emotional factors) how to transfer the gathered information into management and choice of interventions and how to meet the particular patient in his/her context and needs [29, 30].

Using the structured routine described above, all patients with CMP that between Jan 1st 2014 and 20th Nov 2018 (to allow for 1-year follow-up after discharge) that were referred and then admitted to participate in the PT-PRP, were consecutively included in the present study.

#### Rehabilitation and choice of interventions – general remarks

Based on the referral, the examination and the PROMs, the type of interventions used were individualized as for type, number of interventions, frequency and length of the rehabilitation. However, the length of the rehabilitation did not exceed 6 months at the unit since that was the maximum time the patient was allowed to stay before discharged from the rehabilitation-program or to be referred to another facility. The choice of intervention was based on ‘clinical reasoning’ and in accordance with evidence-based medicine and in discussion with the patient, and thus not specifically predefined prior to the start of the program. To create a goal-oriented rehabilitation, each patient in collaboration with the physiotherapist, formulated value-oriented, activity-based goals at start of the rehabilitation, likely to be achieved during the program. The patients’ activity-goals were used for patient motivation, when discussing the choice of interventions and when to decide the end of the program. The PT-PRP also included home-exercises as an essential part of the rehabilitation. Notably, the interventions used in this study were not at the discretion of the researchers, meaning that the interventions were not offered only to this particular study-population, and were delivered according to regular care (thus no ‘Trial registration’ was required for the present study).

#### Categorization of all interventions performed in the PT-PRP

Data on what specific interventions that each patient engaged in during the rehabilitation-program was collected by the first author by reading the medical records for all 274 patients in the cohort using the following manual. The manual was developed from the following steps: *i)* the first author studied the development of treatment categories as described in prior literature of primary care physiotherapy interventions [32], *ii)* these categories were discussed with all physiotherapists at the unit (11 professionals) and 10 categories were agreed upon, *iii)* the first author used the 10 categories to scrutinize five medical records (not used in the present study). The list of categories were adjusted, finally settled, and a manual was written on how to read and categorize all interventions found in the medical records Table 1. Also, the number of patients who were advised on exercises and regimes to perform on their own (at home, at work/school, or in a gym facility) were counted from the medical records.

**Table 1:** Categorization of all interventions used in the program, defined in ten categories

Intervention	Description
1. Education	General education on injury/disease, acute and chronic pain-physiology, neuroscience education, regimes, activity-modification, ergonomics, general health, coping strategies
2. Sensorimotor training	Can also be termed neuromuscular training or motor control exercises and refers to exercises with the purpose of improving sensorimotor control - including training of muscular synergies, balanced in time and magnitude through the relearning of motor control - in specific, the exercises aim at creating appropriate, automatic and generalized movements to optimize muscular joint stabilization commonly affected by the pain [33, 34]
3. Physical activity-advice	Individualized advice on how to optimize the individual patient’s physical activity despite the chronic pain, most often advice on walking, Nordic walking, cross-training or cycling.
4. Interventions aiming at improving structures and functions	Stretching, manual physical therapy techniques, heat or cold treatment, spray-n-stretch-technique, taping – all prior to exercises for movement or stabilization
5. Sensory stimulation	Transcutaneous electrical nerve stimulation, acupuncture
6. Physical activity carried out together with the physiotherapist	Physical activity with direct guidance on for example exercise-bike, cross-trainer or Nordic walking
7. Weight training	Individualized exercises performed in a gym center or weight training with other equipment
8. Relaxation, Mindfulness	Relaxation techniques such as breathing exercises, progressive muscle relaxation, autogenous training. Mindfulness-training including paying attention to thoughts, feelings, bodily sensations other than pain in the present moment, often while carrying out movements
9. Physiotherapy interventions with an Acceptance and commitment therapy (ACT)-inspired approach	Interventions to support acceptance, committed action, values, movement- and behavior change and to reduce fear-of-movement, and also specific interventions on motivation - MI, motivating interviewing - aiming at improving self-efficacy in behavior-change
10. Basic body awareness therapy and training of specified activities	A movement-based physiotherapeutic method developed in Scandinavia where movements aim at enhance body awareness and consciousness of the body with the purpose to move with less effort [35]

#### Physiotherapists

The physiotherapists, a total of 11 Registered Physical Therapists involved in the PT-PRP during the five years of the study, were all trained in Sweden and with particular formal qualifications and expertise on assessment and rehabilitation in acute and chronic pain, orthopedics, Orthopedic Manual Physical Therapy, sensorimotor control and on rehabilitation and specific interventions in patients with CMP. All physiotherapists had 2016–2018 taken part in an education program at the unit, aiming at including also an approach inspired by the tenets of Acceptance and Commitment Therapy (ACT), in the program, for example by emphasizing a value-oriented perspective and committed actions based on the patients’ values in goal-setting, using mindfulness as an intervention for contact with the present moment, and also, when appropriate, focus on the improvement of value-oriented functions over reducing pain. Two physiotherapists had PhD degrees allowing for research competence.

#### Assessments and questionnaires – main clinical outcomes

The patients answered questionnaires at the start and at discharge of the rehabilitation-program (or after a maximum of 6 months) and at 1 year after discharge of the rehabilitation-program (1-year follow-up). The primary outcome was: perceived pain during last week on Numeric Pain Rating scale (NPRS) [36], 0–10 (0 corresponding to no pain, 10 corresponding to worst pain imaginable), and the two secondary outcomes were: 2) ratings of perceived disability on Disability Rating Index (DRI - a visual analogue scale 0–100, 0 corresponding to “no disability”, 100 corresponding to “cannot carry out” (the founder describes the following expressions to correspond to the DRI-points: mild disability = 25, moderate disability = 50, severe disability = 75, 100 = cannot carry out) in 12 activities; dressing, walking, stair climbing, sitting down, leaning over a zinc, carrying a bag, making the bed, running, easy manual labor, heavy manual labor, heavy lifting and sports/exercise) [37, 38] and 3) ratings of perceived overall health status according to EQ-5D, EuroQol five diemsnion scale, [39] where EQVAS (the rating of perceived health on a scale from 0=“worst health imaginable”, to 100=“best health imaginable”) was used.

#### Global assessments of pain management

In addition, global assessments at start, at discharge and at 1-year follow-up of the patients’ experienced ability in reducing pain/affliction were recorded: “What is your opinion on your ability to reduce your pain/ailment?” (0–6, 0 = not at all, 6 = high ability), and also one question used in the Swedish Quality Registry for Pain Rehabilitation, SQRP [40]: “Has your rehabilitation influenced your ability to manage overall life circumstances?”, 1–5, 1 = much worse, 2 = worse, 3 = no change, 4 = improved, 5 = much improved, [40]. Moreover, the EQ-5D separate question on rather/very/extreme anxiety/depression (representing option “3″ on the EQ-5D 3-level option - used in the early years of data collection - and option “3, “4″ or “5″ on the EQ-5D 5-level option - used in later years of the study) was used [41, 42].

#### Global assessments of physical activity

Two questions were used to monitor the amount of weekly physical activity. At start, at discharge and at 1-year follow-up the patients answered: 1) “For how long do you practice leisure exercise per week, for example walking, biking or gardening? Count the total sum of minutes spent (minimum 10 min/interval)”: this was categorized as “less than 30 min/week”, “30–60 min/week”, “60–90 min/week”, “90–150 min/week”, “150–300 min/week”, “more than 300 min/week” (question according to Swedish National Institute of Public Health [43]), and 2) “In your opinion, has your rehabilitation changed your ability to be physically active (for example in daily activities, walking, exercise/training)?”, 1–5, 1 = much worse, 2 = worse, 3 = no change, 4 = improved, 5 = much improved.

#### Data analysis and statistical methods

The study was a longitudinal cohort-study and descriptive statistics were used to analyze frequencies and distribution. The interventions used are presented in proportions (%) and a cluster analysis was performed to calculate the most common combination of interventions. Moreover, clinical outcomes such as the patients’ ratings of pain, disability and health status at start, discharge and at 1-year follow-up were calculated. The following minimal clinical important differences (MCID) for improvement were used; NPRS, minimum two points [44, 45], disability in activity (as measured on DRI) minimum 10% [46], and perceived health status (EQVAS), minimum 20% [39]. Since the measurements were considered to be ordinal scale data, median, minimum – maximum, quartiles and non-parametric statistics were used in analyses. Within-group comparisons were calculated with non-parametric statistics (Wilcoxon signed rank test), and between-group comparisons were tested with the Mann-Whitney U-test. For all comparisons, differences *p* ≤ 0.01 were considered statistically significant. All calculations were performed using IBM SPSS Statistics 25.

The study was approved by Swedish Ethical Review Agency (Dnr: 2019–03701).

## Results

**Fig. 1:**
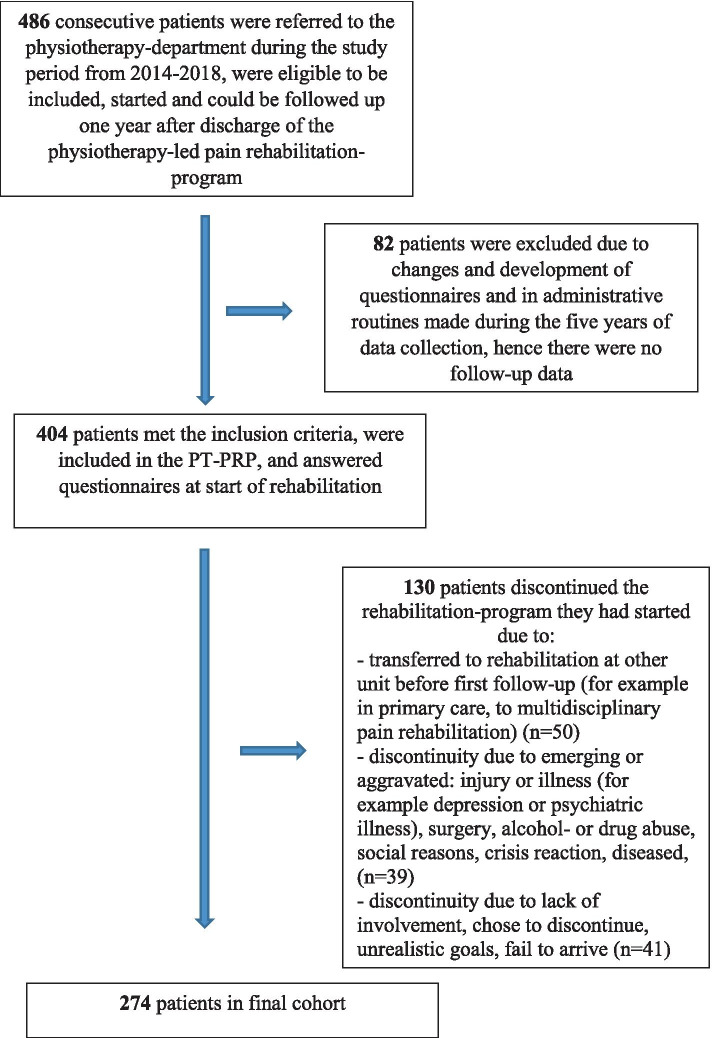
Flow chart of consecutive inclusion and exclusion of patients in the Physiotherapy Pain Rehabilitation Program, PT-PRP, during the data collection period 2014–2018

During the study period Jan 1st 2014 to 20th Nov 2018, a total of 486 consecutive patients were referred to the physiotherapy department and eligible to be included. All individuals that started the rehabilitation program and were included at a date that allowed for follow-up at discharge and at 1-year after discharge in the PT-PRP were included. Due to administrative changes and the development of the questionnaires for clinical purposes, 82 patients were excluded because of changes in the early versions of the questionnaires, and hence there were no follow-up data for these patients. Also, during the almost 5 years of the study, 130 patients discontinued or postponed their rehabilitation-program due to for example new injury, surgery or developed new illness that was not known at inclusion, Fig. 1. Therefore, in the final cohort, 274 patients were included. For patient characteristics see Table 2.

**Table 2:** Characteristics of the study population of 274 patients at start of the rehabilitation-program

Age, mean ± SD (min-max), years	42 ± 13.4 (18–77)
Women, n (%)	194 (71)
BMI, mean ± SD, kg/m^2^	25.0 ± 4.1
Smokers, n (%)	21 (8)
Pain-duration, median [q1, q3], days	1015 [516, 2514]
Pain distribution as diagnosed by physiotherapist, n (%), valid *n* = 265	
- localized pain	104 (39)
- regional pain	145 (55)
- generalized pain	16 (6)
Main pain location, n (%), valid *n* = 270	
- neck region	76 (28)
- cannot decide on only one location as the most painful region	65 (24)
- lumbar- or thoracic spine- or pelvis region,	60 (22)
- hip-, knee-, ankle- or foot region	47 (17)
- shoulder region, elbow- or hand region	22 (8)
NPRS^1^ inclusion, median [q1, q3]	7 [5, 8]
DRI^2^, median [q1, q3], mm	49 [31, 62]
EQVAS^3^, median [q1, q3], mm	50 [35, 70]
Low ability to reduce pain^4^, n (%)	114 (42)
Physically active^5^ minimum 150 min/week, n (%) valid *n* = 134	48 (36)
Anxious or depressed (EQ5D-5 L^6^ or EQ5D-3 L^6^), n (%)	62 (23)
Number of individual rehabilitation-goals formulated at start^7^, median [q1, q3]	2 [2, 3]

1 = NPRS (Numeric Pain Rating Scale) 0–10; 0 = no pain, 10 = worst pain imaginable, 7 = severe pain, 2 = DRI (Disability Rating Index, mean mm of physical disability of 12 activities) 0–100, 0 = no disability, 100 = cannot at all carry out, 3 = EQVAS (EuroQol five dimensions questionnaire, visual analogue scale), 0–100, 0 = worst perceived health imaginable 100 = best perceived health imaginable, 4 = Rated ability to reduce pain = 0–2 on a scale of 0–6; 0 = cannot reduce pain at all, 6 = high ability to reduce pain, 5 = question according to Swedish National Institute of Public Health; “For how long do you practice leisure exercise per week, for example walking, biking or gardening?” (missing *n* = 140 due to that the question was not added until 2017), 6 = EQ5D-5 L and EQ5D-3 L EuroQol five dimensions questionnaire with 5 and 3 levels respectively. 7 = number of value-oriented, activity-based goals formulated at start of the rehabilitation by patient and physiotherapist and likely to be achieved during the program

### The interventions

The four interventions practiced by most patients in the PT-PRP were education, sensorimotor training, physical activity-advice and interventions aiming at improving structures and functions, as demonstrated in Table 3. The most common combination of interventions was: education, sensorimotor training and interventions aiming at improving structures and functions and was practiced by 138 patients, 50%, Table 4. The median number of sessions for the PT-PRP was nine [q1 = 5; q3 = 14], with a median duration of 5 months, and each patient participated in a median of 4 different interventions [q1 = 4; q3 = 5]. Also, the medical records showed that 99% of the patients were advised on exercises and regimes to perform on their own at home, at work/school and/or in a gym facility.

**Table 3:** Percentages of the whole population, *n* = 274, that participated in the listed interventions during the rehabilitation-program

Interventions	%
1. Education	97.4
2. Sensorimotor training	91.2
3. Physical Activity-advice	67.9
4. Interventions aiming at improving structures and functions	67.2
5. Sensory stimulation	50.0
6. Physical Activity performed together with the physical therapist	22.3
7. Weight training	16.8
8. Relaxation, Mindfulness	16.1
9. Physiotherapy with an ACT-inspired approach	12.8
10. Basic body awareness therapy and training of specified activities	9.5

**Table 4:** Final clusters showing the most common interventions in the rehabilitation-program

Interventions	Cluster		
	1	2	3
1. Education	1	1	1
2. Sensorimotor training	1	1	1
3. Physical Activity-advice	1	0	1
4. Interventions aiming at improving structures and functions	1	1	0
5. Sensory stimulation	1	0	0
6. Physical Activity performed together with the physical therapist	0	0	1
7. Weight training	0	0	1
8. Relaxation, Mindfulness	0	0	0
9. Physiotherapy-interventions with an ACT-inspired approach	0	0	0
10. Basic body awareness therapy and training of specified activities	0	0	0
Number of cases in Cluster	115	138	21

As calculated with Centers of clusters. Number of cases in cluster; 274 valid. For specific description of interventions see Table 1

### Main clinical outcomes

**Fig. 2:**
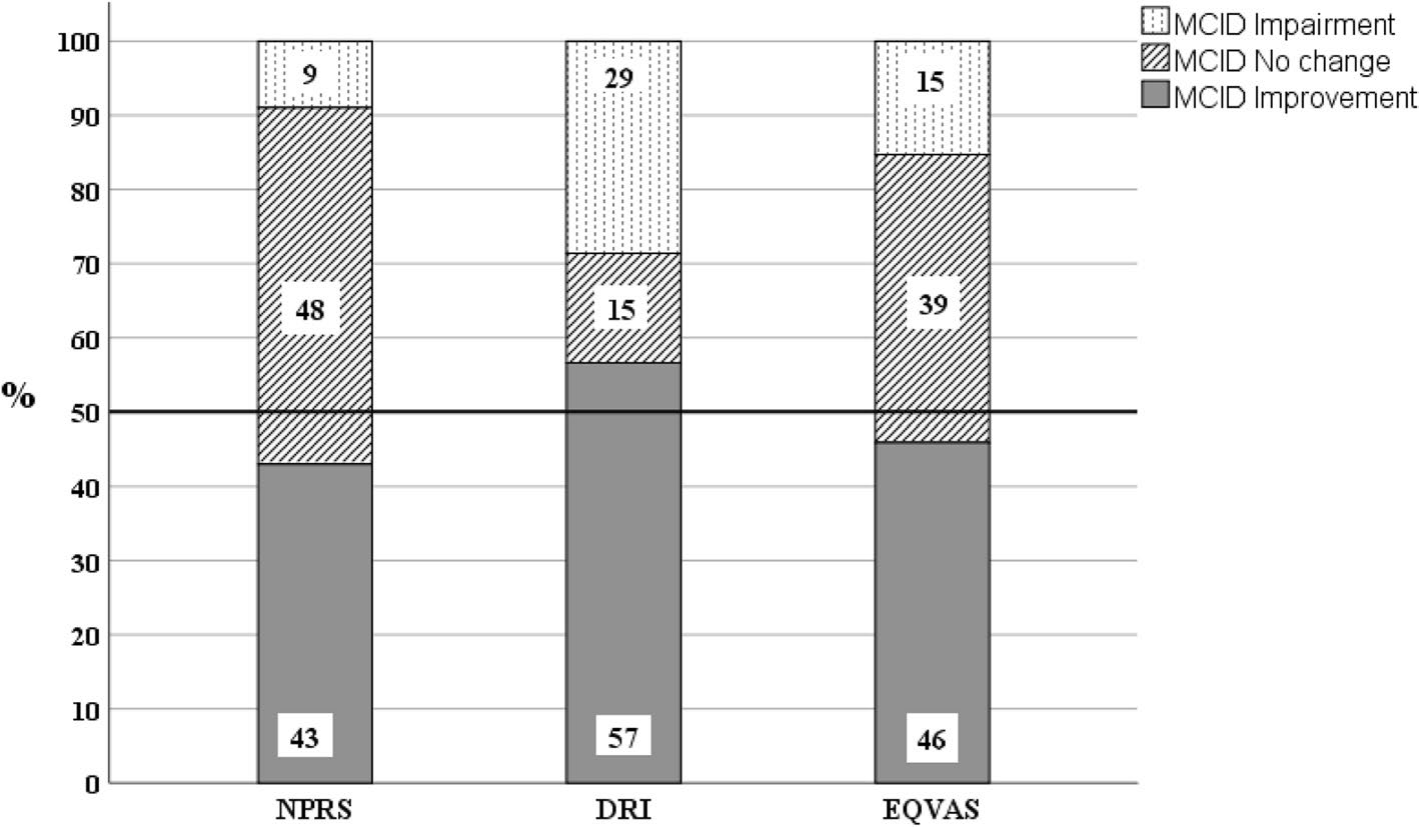
Percent of patients with minimal clinical important difference (MCID) between start and at follow-up 1 year after discharge of the rehabilitation-program PT-PRP, *n* = 178. NPRS=Numeric Pain Rating Scale, DRI=Disability Rating Index (ratings of perceived disability), EQVAS = EuroQol five-dimension VAS scale (ratings of overall health)

The patients’ ratings of NPRS, DRI and EQVAS improved between start and discharge, and between start and 1-year follow-up, *p* < 0.001 respectively, Table 5. Forty-five percent of the patients rated a clinically important improvement of pain between start and discharge and 43% between start and 1-year follow-up. Moreover, 61% had a clinically relevant improvement of disability as rated on DRI between start and discharge, and 57% between start and 1-year follow-up. Additionally, 50% of the patients had a clinically important improvement of perceived overall health status of at least 20% between start and discharge, and 46% between start and 1-year follow-up. One-year follow-up data are presented in Fig. 2.

**Table 5:** Individual changes in ratings of pain, disability and overall health

		Individual change from:			
Ratings of:	Start of program, group level	start to discharge	discharge to 1-year follow-up	start to 1-year follow-up	start to 1-year follow-up, %
NPRS^1^ 0–10 median [q1, q3]	7 [5, 8] *n* = 265	-1 [-3, 0] *p* < 0.001 *n* = 242	0 [-1, 1] *p* = 0.36 *n* = 171	-1 [-3, 0] *p* < 0.001 *n* = 178	-17% [-43, 0] % *n* = 178
DRI^2^, 0–100 median [q1, q3], mm	48 [31, 62] *n* = 256	-6 [-15, 2] *p* < 0.001 *n* = 230	-1.5 [-9, 6] *p* = 0.26 *n* = 168	-6 [-18, 5] *p* < 0.001 *n* = 175	-15% [-44, 13] % *n* = 175
EQVAS^3^,0–100 median [q1, q3], mm	50 [35, 70] *n* = 259	10 [0, 20] *p* < 0.001 *n* = 232	0 [-10, 10] *p* = 0.82 *n* = 164	7.5 [-5, 25] *p* < 0.001 *n* = 170	14% [-7, 50] % *n* = 170

Ratings of pain, disability and overall health at start of the rehabilitation-program PT-PRP and median of individual changes in ratings from start to discharge of program, from discharge to 1-year follow-up and from start to 1-year follow-up (one year after discharge of the rehabilitation-program)

1 = NPRS (Numeric Pain Rating Scale) 0–10; 0 = no pain, 10 = worst pain imaginable, 2 = DRI (Disability Rating Index, mean mm of physical disability of 12 activities) 0–100, 0 = no disability, 100 = cannot at all carry out, 3 = EQVAS (EuroQol five dimensions questionnaire, visual analogue scale), 0–100, 0 = worst perceived overall health imaginable 100 = best perceived overall health imaginable. For details see Methods. Note that for the NPRS- and DRI-scales, a higher rating means worse outcome, and the reverse for EQVAS

### Global assessments

At start of PT-PRP, only 11% of the patients rated a high ability to reduce pain/ailment (alternative 5 and 6 on the scale from 0 to 6). At discharge of the rehabilitation-program this was improved to 34%, *p* < 0.001, and was reduced to 26% at 1-year follow-up but was still improved when compared to start, *p* < 0.001 Table 6. The majority of the patients, 85%, rated that the rehabilitation had improved or much improved their ability to better manage life circumstances overall at discharge of the rehabilitation-program. At 1-year follow-up, there was no significant reduction in improvement (*p* = 0.367), and 74% of the patients still rated that the rehabilitation had improved or much improved their ability to manage life circumstances. There was also a statistically significant improvement in the proportion of patients rating a rather/very/extreme anxiety or depression, 23% at start vs 16% at 1-year follow-up, *p* = 0.006 Table 6.

**Table 6:** Results of global assessments

Global assessment question	Start	Discharge	1-year follow-up
1. Percent rating high ability (alternative 5 or 6 on a scale 0–6): “What is your opinion on your ability to reduce your pain/ailment?”	11%	34% *p* < 0.001, as compared to start	26% *p* < 0.001, as compared to start
2. Percent rating “improved” or “much improved”: “Has your rehabilitation influenced your ability to manage overall life circumstances?”	–	85%	74% *p* = 0.367, as compared to discharge
3. Percent rating the EQ-5D separate question on rather/very/extreme anxiety/depression	23%	13% *p* < 0.001 as compared to start	16% *p* = 0.006 as compared to start
4. Percent scoring physical leisure exercise of minimum 150 min or more/week (for example walking, biking or gardening, count the total sum of minutes spent)	36%	44% *p* = 0.009 as compared to start	43% *p* = 0.001 as compared to start
5. Percent scoring “improved” or “much improved”: “In your opinion, has your rehabilitation changed your ability to be physically active (for example in daily activities, walking, exercise/training)?”	–	76%	69% *p* = 0.775 as compared to discharge

2 = question also used in the Swedish Quality Registry for Pain Rehabilitation, SQRP [40], 3 = the separate question from EQ-5D on rather/very/extreme anxiety/depression (representing option “3” on the EQ-5D 3-level option - and option “3, “4″ or “5″ on the EQ-5D 5-level option [41, 42], 4 = question according to Swedish National Institute of Public Health [43] “For how long do you practice leisure exercise per week, for example walking, biking or gardening? Count the total sum of minutes spent (minimum 10 min/interval)”, 5 = “In your opinion, has your rehabilitation changed your ability to be physically active (for example in daily activities, walking, exercise/training)?”

The patients’ ratings of physical leisure exercise of minimum 150 min or more/week improved from 36% at start, to 43% at 1-year follow-up (*p* = 0.001). Also, most of the patients, rated at discharge of the program that the rehabilitation had improved their ability to be physically active (in for example daily activities, walking, exercise/training) and at 1-year follow-up, there was no significant reduction in improvement (*p* = 0.775), since still 69% of the patients rated that the rehabilitation period had improved their ability to be physically active Table 6.

Pain ratings were completed by 178 patients both at start and at 1-year follow-up, meaning that 96 patients had not filled out the specific pain-rating-question in the 1-year follow-up-questionnaire. There were no differences in baseline measurements of primary and secondary outcomes at start of the rehabilitation-program for this sub-group of 96 patients as compared to the group of 178 patients: NPRS median = 7 [q1 = 5; q3 = 8], *p* = 0.655, DRI median = 45 [q1 = 26; q3 = 60] mm, *p* = 0.228 or in EQVAS median = 50 [q1 = 30; q3 = 70] mm, *p* = 0.309. See also Table 5.

At discharge, the number of rehabilitation goals formulated for each patient at start as likely to be achieved during the program, and the number of goals that in fact were reached, were calculated, showing a median goal achievement of 75% [q1 = 50, q3 = 100].

## Discussion

This study investigated the interventions used in a one-to-one, physiotherapist-led rehabilitation-program for a sub-group of patients with CMP in specialized care. The results showed that the main interventions were education, sensorimotor training, physical activity-advice and interventions aiming at improving structures and functions. Furthermore, although all patients had, prior to this rehabilitation program, participated in physiotherapist-led interventions and were deemed refractory to further improvement by the referring caring facility, about half of them reported clinically important improvements on pain, disability and overall health at discharge and 1-year after discharge of this resource-effective rehabilitation-program of median nine sessions during five months, monitored only by one physiotherapist.

### Discussion on interventions

Education and sensorimotor training were the two interventions most often used in the present study. This is in line with the recommendations on non-pharmacological management of chronic pain in guidelines and in prior studies, supporting individualized education and communication along with supervised, individualized group exercises and physical activity [4, 14, 18]. The results are also in line with Wijma et al. advocating pain neuroscience education explaining the neurophysiology and biopsychosocial interaction in chronic pain [22]. Yet, also in primary care settings, it has been described that advice and exercise therapy were the most frequent interventions in patients with non-specific, subacute low-back-, neck- or subacromial pain [47]. It was not unexpected that the current PT-PRP to some extent used similar interventions as in primary care settings, but the education in the PT-PRP also consisted of education on pain neuroscience, chronic pain-physiology and chronic pain management, shown not only to improve patients’ knowledge of pain, but also to reduce pain, disability, catastrophizing, and to enhance physical performance [48].

Sensorimotor training was the second most used intervention used in the present study. The terms “exercise therapy” and “physical exercise” are not completely equivalent to the term “sensorimotor training” used in the present study, but analogous. The term “sensorimotor training” used here describes training of fundamental prerequisites, aiming at optimizing muscular joint stabilization affected by the initial pain, by the training of muscular synergies, through the relearning of motor control [33, 34]. This training can easily be individualized and adjusted also for patients with severe pain and kinesiophobia, and can be used before and in parallel to more heavy training, for example strength training. These exercises are also in line with what is suggested by Booth et al. describing “consensus for individualized, supervised exercise based on patient presentation, goals and preference that is perceived as safe and non-threatening to avoid fostering unhelpful associations between physical activity and pain.” [28] in patients with CMP.

The results also showed that physical activity-advice was offered to almost 70% of the patients. It is currently well established that physical activity can be beneficial for patients with CMP [14, 28, 49, 50] and tailored exercises to individual patients have been recommended [51] although no evidence exist on what particular type of exercise is the most beneficial for these patients [51, 52]. According to available evidence, all patients should be offered advice on physical activity, and it was therefore expected that all individuals participating in this program should have been offered this. However, in the medical records, the word “information” or “education” was sometimes used with no further specification on the content of what was informed about. According to the pre-set manual used to categorize interventions in the study - to be categorized as having received advice on physical activity, these precise words had to be clearly stated in the medical records, and it is therefore not unlikely that an even higher proportion than 70% did indeed receive advice on physical activity.

Interventions aiming at improving structures and functions were practiced on almost as many patients as physical activityadvice in the PT-PRP. The evidence for this kind of treatment in patients with CMP is low, used as a sole intervention [24]. It should be noted, that in the present study interventions for structures and functions were commonly used in combination with other interventions (actually in the most common combination), involving education and sensorimotor training, Table 4 [25].

An intervention with ACT-approach was described in the medical records for only 12.8% of the patients despite that the physiotherapists had taken part in an educational course at the unit, with the purpose to integrate an ACT-approach into the rehabilitation. This low outcome from behavioural medicine interventions is in line with prior studies of similar type of rehabilitation but in a different setting, showing even lower results for a patient-group with acute/subacute pain but with established psychological- or social and environmental risk factors [32]. One reason for the low outcome in the present study can be due to the known difficulties in implementing new treatments [53]. Also, personal communication after the data collection have revealed that sometimes the physiotherapists did not use the “ACT-vocabulary” in the medical records, but instead used the word “information” or “education” with no further specification, to describe discussions on for example acceptance of chronic pain and subsequent limitations, in discussions on committed actions, in goal-setting according to values, in discussions on motivation, or in behaviour changes and achievements to reduce fear of movement.

There is no consensus on the optimal length, content or intensity of rehabilitation in patients with CMP [17], which allows for the establishment and assessment of alternative set-ups of rehabilitation-programs, as the one presented here. The PT-PRP was individualized regarding interventions, frequency and length and involved a goal-oriented teamwork between the patient and one physiotherapist. This we regard as a favorable set-up for patient-centered care, which has been proposed to be a cornerstone in the rehabilitation of musculoskeletal pain in primary care [24]. However, if the patients did not develop further detrimental consequences due to additional pain related complaints, the set-up of the PT-PRP could be considered as a rehabilitation option for a subgroup of patients with CMP referred due to exhausted treatment resources in for example primary care but still not in the need of extensive, costly, interdisciplinary pain rehabilitation. We therefore find it highly important to identify what characterizes this subgroup of patients that did benefit from this mode of rehabilitation compared to those who did not. Such a study is now in process.

### Discussion on clinical outcomes

The program-goals of clinical outcomes on pain, activity and overall health at 1-year follow-up after the PT-PRP show promising results, especially when considering that the individuals were referred due to being refractory to preceding treatments at the referring caring facility. To the best of our knowledge, no similar studies with focus on rehabilitation of patients with complex, persisting pain problems are to date presented in the literature, therefore comparisons to other studies are challenging. Yet, small but significant effects in terms of reduced pain were observed in a meta-analysis conducted by Searle et al. when pooling the results across 39 RCT’s covering 4462 participants after strength/resistance- and coordination/stabilization-exercise programs in patients with chronic low-back pain [54]. Bertozzi et al. conducted a meta-analysis comprising seven RCT’s on patients with chronic nonspecific neck pain, and found medium, significant overall effect size in reducing pain, and median but not significant overall effect size in reducing disability one to 6 months after therapeutic exercises [55]. However, no long-term results could be calculated due to lack of studies assessing follow-up longer than 6 months [55]. It is also worth noticing that in an overview of Cochrane reviews by Geneen et al. 2017, mostly small-to-moderate effects on pain severity and improved physical function were found in patients with chronic pain at follow-up at three to 6 months after interventions consisting of physical activity and exercise [4]. All in all, when comparing the results in the present study with the studies described above, our results 1 year after discharge of the PT-PRP, can be considered encouraging, and the PT-PRP seems to be a resource effective option (comprises only one professional in the team with the patient) for this sub-group of patients with chronic severe pain and disability.

Reflections on the uniqueness of the PT-PRP and the prerequisites to deliver such a program can be made from a clinical point of view. One corner stone might be the advanced knowledge and experience held by the physiotherapists in the PT-PRP in the examination and rehabilitation of *both* curable conditions of acute pain and a pain-contingent treatment approach as well as of CMP and a time-contingent treatment approach, and that the different interventions used could be implemented simultaneously and be performed in parallel by the same physiotherapists at the same caring facility. Further, working with value-oriented goals and the physiotherapists’ experience of patients with CMP and from working in teams with other professionals (physician, psychologist) at the specialized pain-unit, can be a key factor. Also valuable is the extended one-to-one time offered for each session (which may be more often possible to offer within specialized care), according to structured methods that are in line with accredited standards [26]. The close access to colleagues at the pain unit for consultation or further referral, together with the considerations above, can be important for the physiotherapists to be perseverant in interventions (meaning that exercises/interventions were upgraded slowly using non-threatening and motivating exercises to avoid unnecessary associations between physical exercise and pain as described by [28], or when this approach was not successful, in striving for value-based activities, the patient was encouraged to explore continuation of interventions despite an increase in symptoms - however not if there was a risk for tissue damage or bodily harm, as in for example neuropathic pain). It should however be noted, that the PT-PRP was directed to a sub-group of patients with less complex consequences, deemed to benefit from defined physiotherapy regime and not for patients with more profound consequences of their pain, and that the rehabilitation attempts made at the referring caring facility until refractory to further improvement, can be a prerequisite paving the way for motivation for treatment-change and for acceptance of chronic pain.

### Discussion on methods and limitations

In the present study we have performed many comparisons, and to avoid chance findings due to multiple testing we conservatively set the significance level at *p* ≤ 0.01.

We used the medical records to identify interventions used in the rehabilitation-program. This method is not validated, and the difference between writing a medical record for medical purposes as compared to using and collecting this data for scientific purposes could have influenced the data collected. Also, this procedure could involve a risk for miss-classification as described above; for example could the category “education” could have been used in a broad perspective in the medical record (especially when used with no other specification), containing interventions that could have been categorized elsewhere. On the other hand, this procedure avoided the problem discussed by Forsbrand et al. who concluded that “self-reporting” of intervention-data into a predefined protocol did not fully reflect the actual behavior of what interventions were performed [32]. Another advantage with our method was that the reading of the medical records and the categorization was done in the exact same way since one and the same person read all records and categorized all data, also resulting in no missing data for any patient. But the fact that the interventions were not predefined but individualized as for type, frequency and length and therefore could be different from one patient to another, can be a limitation when considering reproducing the procedure. To meet the demand for consensus on the optimal length, content or intensity of rehabilitation in patients with CMP [17], it is recommended that descriptions of procedures and content in future studies are made in detail, although this require clear definitions and structure also in clinical practice.

The number of patients excluded due to changes in data collection (82 patients) and the number of patients that chose to discontinue the program (130) during the 5 years of data collection are a limitation of the study to be taken into account. Further, there was no comparison group to the clinical data, since the main aim of the study was to describe the interventions used in the rehabilitation-program. Still, we argue that since the patients had been refractory to preceding treatments at the prior caring facility with persistent complaints, they could be regarded as their own control.

At program inclusion, decision making was based on structured ‘clinical reasoning’ (using the referral, examination and on patient-relevant outcome measures), but no predefined, criteria developed for scientific reasons were used during this process since this was part of regular, clinical practice. This is a limitation of the study, especially when considering reproducing the procedure, and the exact decision-making criteria used, made by the physiotherapist in collaboration with the patient, remains to be addressed in a forthcoming study.

We are aware that employment or working status is a highly important factor in chronic pain [56, 57] and it would have been a strength of the study if we had been able to present this information. Unfortunately the questionnaires used were altered several times over the years of the study and no consistent data on employment was obtainable. It would also have been a strength if data on pharmacological pain-treatment were assessed at start and follow-up, but no such data was available in the PROMs used in the program focusing on a physiotherapist-led interventions. Any effects of such medication, both positive and negative, are thus included in the patients’ ratings of their perceived pain both at start and at follow-up, along with all other interventions and circumstances in life that may effect their rating of experienced pain.

In conclusion, for half of the patients, referred due to refractory to preceding treatments at the prior caring facility and with persistent complaints of CMP, a combination of individualized education, sensorimotor training, physical activity-advice and interventions for structures/functions rendered promising clinically important long-term improvements on pain, disability and overall health after only nine, one-to-one physiotherapist-led rehabilitation sessions.

## Data Availability

The data that support the findings of this study are available from Region Skåne but restrictions apply to the availability of these data since it is based on medical records, which were used under license for the current study, and so are not publicly available. Data are however available from the authors upon reasonable request and with permission of “The Commission of quality registry for caring-databases at Region Skåne (KVB)” (after approval of “Application for disclosure of personal information from the Region Skåne databases and quality registries”) and after approval of the Swedish Ethical Review Agency, Etikprövningsmyndigheten.

## Abbreviations

ACT: Acceptance and commitment therapy
DRI: Disability Rating Index
EBM: Evidence Based Medicine
EQVAS: EQ-5D, EuroQol five-dimension Visual Analogue Scale
MCID: Minimal Clinical Important Differences
NPRS: Numeric Pain Rating Scale
PT-PRP: Physiotherapy Pain Rehabilitation Program
RCT: Randomized controlled trial
SQRP: Swedish Quality Registry for Pain Rehabilitation
